# Biofunctional Activities of *Equisetum ramosissimum* Extract: Protective Effects against Oxidation, Melanoma, and Melanogenesis

**DOI:** 10.1155/2016/2853543

**Published:** 2016-06-15

**Authors:** Pin-Hui Li, Yu-Pin Chiu, Chieh-Chih Shih, Zhi-Hong Wen, Laura Kaodichi Ibeto, Shu-Hung Huang, Chien Chih Chiu, Dik-Lung Ma, Chung-Hang Leung, Yaw-Nan Chang, Hui-Min David Wang

**Affiliations:** ^1^Department of Fragrance and Cosmetic Science, Kaohsiung Medical University, 100 Shih-Chuan 1st Road, San Ming District, Kaohsiung 807, Taiwan; ^2^Department of Biotechnology, National Formosa University, 64 Wenhua Road, Huwei Township, Yunlin County 632, Taiwan; ^3^Department of Marine Biotechnology and Resources, National Sun Yat-Sen University, 70 Lianhai Road, Gushan District, Kaohsiung 804, Taiwan; ^4^Department of Biological Science, University of North Carolina at Chapel Hill, Coker Hall, CB No. 3280, 120 South Road, Chapel Hill, NC 27599-3280, USA; ^5^Department of Biomedical Science, Barry University, 11300 Northeast 2nd Avenue, Miami, FL 33161, USA; ^6^Graduate Institute of Medicine, College of Medicine, Kaohsiung Medical University, 100 Shih-Chuan 1st Road, San Ming District, Kaohsiung 807, Taiwan; ^7^Center for Stem Cell Research, Kaohsiung Medical University, 100 Shih-Chuan 1st Road, San Ming District, Kaohsiung 807, Taiwan; ^8^Division of Plastic Surgery, Department of Surgery, Kaohsiung Medical University Hospital, Kaohsiung Medical University, Kaohsiung 807, Taiwan; ^9^Department of Surgery, Faculty of Medicine, College of Medicine, Kaohsiung Medical University, Kaohsiung 807, Taiwan; ^10^Department of Biotechnology, Kaohsiung Medical University, Kaohsiung 807, Taiwan; ^11^Department of Chemistry, Hong Kong Baptist University, Kowloon Tong, Hong Kong; ^12^State Key Laboratory of Quality Research in Chinese Medicine, Institute of Chinese Medical Sciences, University of Macau, Macau; ^13^Graduate Institute of Natural Products, Kaohsiung Medical University, Kaohsiung 807, Taiwan

## Abstract

*Equisetum ramosissimum,* a genus of Equisetaceae, is a medicinal plant that can be separated into ethyl acetate (EA), dichloromethane (DM),* n*-hexane (Hex), methanol (MeOH), and water extracts. EA extract was known to have potent antioxidative properties, reducing power, DPPH scavenging activity, and metal ion chelating activity. This study compared these five extracts in terms of their inhibiting effects on three human malignant melanomas: A375, A375.S2, and A2058. MTT assay presented the notion that both EA and DM extracts inhibited melanoma growth but did not affect the viabilities of normal dermal keratinocytes (HaCaT) or fibroblasts. Western blot analyses showed that both EA and DM extracts induced overexpression of caspase proteins in all three melanomas. To determine their roles in melanogenesis, this study analyzed their* in vitro* suppressive effects on mushroom tyrosinase. All extracts except for water revealed moderate suppressive effects. None of the extracts affected B16-F10 cells proliferation. EA extract inhibited cellular melanin production whereas DM extract unexpectedly enhanced cellular pigmentation in B16-F10 cells. Data for modulations of microphthalmia-associated transcription factor, tyrosinase, tyrosinase-related protein 1, and tyrosinase-related protein 2 showed that EA extract inhibited protein expression mentioned above whereas DM extract had the opposite effect. Overall, the experiments indicated that the biofunctional activities of EA extract contained in food and cosmetics protect against oxidation, melanoma, and melanin production.

## 1. Introduction

Mitochondria, chloroplasts, and peroxisomes produce reactive oxygen species (ROS) through respiration and photosynthesis [1, 2]. Studies suggested that the changes in cellular homeostasis caused by high ROS levels can result in oxidative damage [3]. To avoid ROS oxidative injuries, the defense radical scavenging systems used by the human being were separated into enzymatic and nonenzymatic mechanisms. Antioxidant enzymes and substances could reduce oxidative damage by decreasing production of ROS and radicals [2]. These agents include glutathione and catalase, glutathione reductase, superoxide dismutase, and glutathione peroxidase. Others include *α*-lipoic acid, carotenoids, coenzyme Q10, flavonoids, antioxidative minerals (copper, zinc, manganese, and selenium), and cofactors (folic acid and vitamins A, B1, B2, B6, B12, C, and E). Generally, the above antioxidative materials are applied in synergic ways with each other against various free radical types [1–3].

Malignant melanoma is among the most incursive and life-threatening malignant tumors [4]. Skin cancer can result from exposure to ultraviolet (UV) radiation emitted by the sun and by halogen lamps. Experimental studies of metastatic melanoma are very challenging because systemic treatments are often ineffective and the rapid spread of melanoma cells to retain an intensive property of the cellular spreading which happens later is pathologically confusing [5]. Although melanoma is not a major cause of tumorous symptoms, melanoma is a major cause of death in patients with skin cancer. Treating melanoma is difficult due to its resistance to conventional chemoradiotherapy [6]. No effective therapies for metastatic melanoma are currently available, and effective drugs are urgently needed.

Melanocytes are located in the basal epidermal layer and in the hair follicles. Generally, UV radiation produces pigment by diametric stimulation of melanocytes [7]. Melanin pigmentation has been recognized by many factors; the permeation of sunlight is a well recognized source of melanin pigmentation; specifically, UV radiation causes darkening of the skin and/or sunburn. UV is the most common reason of changes in the visible countenance of human skin. Unusual melanogenesis is a characteristic of many human skin disorders, including abnormal pigmentation, nevi, and melanoma [8].

Many recent studies have investigated the biological functions of natural extracts and their potential applications as health foods, as active ingredients in cosmetics, and as leading compounds in new medicines [9, 10].* Equisetum ramosissimum,* a genus of Equisetaceae, is a medicinal plant administered to treat hemorrhage, urethritis, jaundice, and hepatitis [11]. Although the antioxidant activities of* E. ramosissimum *were identified [12], its biological activities have not been examined. Therefore, this study elucidated the potential protective effects of* E. ramosissimum* extract against oxidation, melanoma, and melanogenesis.

## 2. Materials and Methods

### 2.1. Chemicals and Reagents

Ascorbic acid (vitamin C), 3-(4,5-dimethylthiazol-2-yl)-2,5-diphenyltetrazolium bromide (MTT), L-3,4-dihydroxyphenylalanine (L-DOPA), dimethyl sulfoxide (DMSO), 1,1-diphenyl-2-picrylhydrazyl (DPPH), ethanol, ethylenediaminetetraacetic acid (EDTA), ferrous chloride (FeCl_2_·4H_2_O), ferric chloride (FeCl_3_), kojic acid, methanol, potassium ferricyanide (K_3_Fe(CN)_6_), 3-tert-butyl-4-hydroxyanisole (BHA), and L-tyrosine were purchased from Sigma-Aldrich Company (St. Louis, MO, USA). Dulbecco's modified Eagle's medium (DMEM) and fetal bovine serum (FBS) were obtained from Gibco BRL (Gaithersburg, MD, USA). Other chemical buffers and reagents were purchased at the highest available purity and quality.

### 2.2. Plant Material Extraction and Isolation

Two authors of this study (Dr. Chieh-Chih Shih and Professor Zhi-Hong Wen) prepared the extracts as follows. First, methanol (2.2 L) was requested in two extraction procedures of* E. ramosissimum* from a powder consisting of ground* E. ramosissimum* plant, and the extract was then refluxed for 30 minutes. After a filtering procedure, the extract was concentrated to obtain methanol (MeOH) crude extract, 9.52 g. The crude extract was added to distilled water (200 mL) and then partitioned with* n*-hexane (Hex, 1.95 g), dichloromethane (DM, 0.67 g), and ethyl acetate (EA, 0.26 g), which left an aqueous layer (H_2_O, 6.44 g). All fractions were concentrated, freeze-dried, dissolved in DMSO to obtain a stock solution (500 mg/mL), and then diluted with DMEM to the required concentrations.

### 2.3. Assays of Antioxidant Effects

#### 2.3.1. Reducing Power Assay

Assays of the reducing power of the crude extracts were performed as described in the literature [13]. Briefly, dissimilar concentrations of each extract were blended with 85 *μ*L of 67 mM sodium phosphate buffer (pH 6.8) and 2.5 *μ*L of 20% K_3_Fe(CN)_6_. The admixture was kept at 50°C for 20 min. After addition of 160 *μ*L 10% TCA, the admixture was centrifuged for 10 min at 3,000 g. The supernatant (75 *μ*L) was mixed with 2% FeCl_3_ (25 *μ*L), and absorbance was read with a spectrophotometer (BioTek Co., Winooski, VT, USA) at 700 nm with a BHA solution as a positive control. High absorbance was interpreted as a high capacity for metal ion reduction.

#### 2.3.2. DPPH^•^ Radical Scavenging Ability Assay

DPPH is a stable free radical with a violet color. Reaction of DPPH^•^ with an antioxidant provides hydrogen, which results in decreased absorbance at 517 nm. DPPH assay was performed as described previously, with some minor modification [13]. Various concentrations of* E. ramosissimum* extracts were added to 100 *μ*L of aqueous stable DPPH^•^ (60 *μ*M) solution and allowed to stand at room temperature for 60 min. Vitamin C was used as a positive control. Low absorbance was interpreted as a high DPPH scavenging ability. The calculation for free radical scavenging activity (%) is done as follows:

(1) $$\begin{array}{c} \text{Scavenging activity}\;\left(\;\%\;\right)=\frac{A_{control}-A_{sample}}{A_{control}}\times 100\%. \end{array}$$

#### 2.3.3. Metal Chelating Activity

The chelating properties of ferrous ions (Fe^2+^) were explored using a method described previously [14]. Briefly, various concentrations of extract were dissolved in DMSO and added to a 10 *μ*L solution of FeCl_2_·4H_2_O (2 mM). Next, 20 *μ*L ferrozine (5 mM) was added, and the admixture was shaken vigorously for 10 minutes. The absorbance was 562 nm. EDTA was used as a positive control, and the formula employed to calculate metal chelating activity is as follows:

(2) Metal  chelating  activity %=Acontrol−AsampleAcontrol×100%.

### 2.4. Cell Line Cultures

Human and rat melanoma cell lines were obtained from Bioresource Collection and Research Center (Taiwan): A375 (BCRC number 60039), A375.S2 (BCRC number 60263), A2058 (BCRC number 60240), and B16-F10 (BCRC number 60031). Human fibroblasts were separated from the foreskin primary culture (Institutional Review Board, KMUH-IRB-990269). Cells were cultured in DMEM supplemented with 10% FBS and 1% antibiotics. Human skin keratinocytes, HaCaT cells, were cultured in Keratinocyte-SFM (Gibco, USA) supplemented with bovine pituitary extract and human recombinant epidermal growth factor. All cell lines were cultured in 5% CO_2_ at 37°C.

### 2.5. MTT Assay of Cell Viability

The influences of extracts on cell development were estimated with MTT assay [15]. Cells were seeded at 8 × 10^3^ cells/well in 96-well plates and incubated for 24 h before addition of extracts. After 24 h, MTT solution was dispensed into each well. After 2 h, the culture medium was discarded, and DMSO was added to each well. The absorbance of the formazan salt was 595 nm, and the cell viability was computed as follows:

(3) $$\begin{array}{c} \text{Cell viability}\;\left(\;\%\;\right)=\frac{A_{control}-A_{sample}}{A_{control}}\times 100\%. \end{array}$$

### 2.6. Western Blot Analysis

This analysis was performed as described previously with some minor modifications [16]. 1 × 10^6^ cells were treated with extracts or with the vehicle control for 24 h, and the cells were then harvested and lysed with RIPA lysis buffer. Equal amounts of protein were separated by sodium dodecyl sulfate-polyacrylamide gel electrophoresis and next transferred into a polyvinylidene fluoride membrane. The membranes were incubated with corresponding primary antibodies and afterwards incubated with secondary antibodies corresponding with the primary antibodies. The signals were visualized with a chemiluminescence detection kit (Amersham, Piscataway, NJ, USA).

### 2.7. Mushroom Tyrosinase Measurement

Mushroom tyrosinase activity was evaluated as described previously with some minor modifications [17]. Samples were incubated with mushroom tyrosinase (25 U/mL), and L-tyrosine (2 mM) in phosphate buffer (pH 6.8) was added. The mixtures were then kept at 37°C for 30 minutes. Kojic acid was used as a positive control. Tyrosinase inhibitory activity was determined by the following equation:

(4) Mushroom  tyrosinase  inhibition %=A−B−C−DA−B×100%,

where *A* is the optical density (OD_490_) without testing extract; *B* is OD_490_ with tyrosinase and without testing extract; *C* is OD_490_ with testing extract; and *D* is OD_490_ with tyrosinase and without testing extract.

### 2.8. Melanin Quantification Assessment

This analysis was demonstrated as described in the literature with some minor modifications [17]. Cell pellets were liquefied with 1.0 N NaOH, warmed to 80°C for 1 hour, and centrifuged at 10,000 g for 10 minutes. The quantity of melanin was determined by extracting the supernatant and running it through the spectrophotometer, which gave a result of 475 nm.

### 2.9. Statistical Analysis

Biofunctional assays of the* E. ramosissimum* extracts in each platform were performed in triplicate. Results were expressed as means ± SD. Analysis of variance was used for data analysis. A *p* value less than 0.05 was considered statistically significant.

## 3. Results and Discussion

### 3.1. Antioxidant Activity of* E. ramosissimum*

Free radicals have roles in signal travel and in physiological, metabolic, and immune reactions. Even though free radicals are needed for normal healthy biochemical processes in the body, they have severe negative health effects [18, 19]. One of the study aims was to test antioxidative properties, which were examined by ferric reducing powers, DPPH radical scavenging capacities, and metal chelating power activities.

First, a simple, rapid, and reliable test was used to measure Fe(III)-ferricyanide complex synthesis. In this test, the extracts reducing properties of* E. ramosissimum* were indicated by changes in the color of the solution (from light yellow to different shades of green and blue).  Table 1 presented the notion that the reducing power of EA extract resulted in stronger dose-dependent suppressive effects compared to the other four extracts and showed the highest scavenging of 0.87 ± 0.23 at 200 *μ*g/mL.

The second oxidation inhibitory assay was DPPH radical scavenging test. As antioxidants stabilize DPPH radicals, the color of DPPH solution changes from violet to yellow as diphenylpicrylhydrazine is formed.  Table 1 illustrated the results for the five extracts, and the comparisons presented the notion that the EA extract had the strongest radical scavenging effects in a dose-dependent manner.

Within the oxidative conditions, ferrozine can develop complexes with Fe^2+^ to be measured quantitatively. In the presence of chelating materials, complex constructions are dislocated, which lightens the red color of the complex.  Table 1 demonstrated the data for the last evaluation of antioxidative properties. The extracts showed low-to-moderate Fe^2+^ scavenging activities at concentrations of 50–200 *μ*g/mL, and EA extract possessed the highest value of 44.56 ± 1.32 at 200 *μ*g/mL.

### 3.2. Cytotoxicity of* E. ramosissimum* on Human Melanoma Cells and Normal Cells

**Figure 1:**
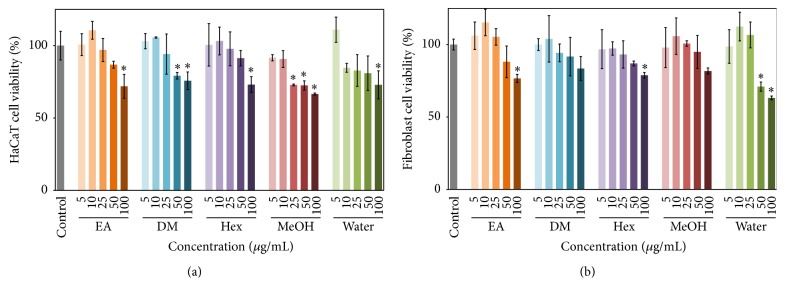
Effects of* E. ramosissimum* extracts on viability of normal human cells according to MTT assay. Suppression of cell viability was measured in (a) HaCaT and (b) fibroblast cells cultured with 5, 10, 25, 50, and 100 *μ*g/mL EA, DM, Hex, MeOH, and water extracts. The results for the control group cultured without extracts were shown on the left (gray line). All experimental data were presented as average values ± SD; *n* = 3; ^*∗*^ *p* < 0.05.

Melanoma is a malignant tumor that starts in a certain type of skin cell and is activated when the abnormal cells in the affected part of the body begin to proliferate in an uncontrolled manner [20]. Metastatic malignant melanomas are highly resistant to existing therapies and have a very poor prognosis, and thus new treatment strategies are urgently needed. In early stages of the development of new chemoprotective substances, the main considerations are normal cell allergic responses, sensitivity and potential reactions, and toxic side effects [9]. MTT method was applied to evaluate the cytotoxic effectivenesses of* E. ramosissimum* extracts on normal human skin cells, including epidermal keratinocytes (HaCaT) and dermal fibroblasts in Figure 1. These two cells were treated with various concentrations (0 to 100 *μ*g/mL) to compare dose-dependent impacts. In both cells, high doses (100 *μ*g/mL) of the five* E. ramosissimum* extracts had minor effects, and all cellular viabilities exceeded 65% after a 24-hour treatment. That is, the* E. ramosissimum* extracts had no severe discernible toxic effects on human normal cells.

**Figure 2:**
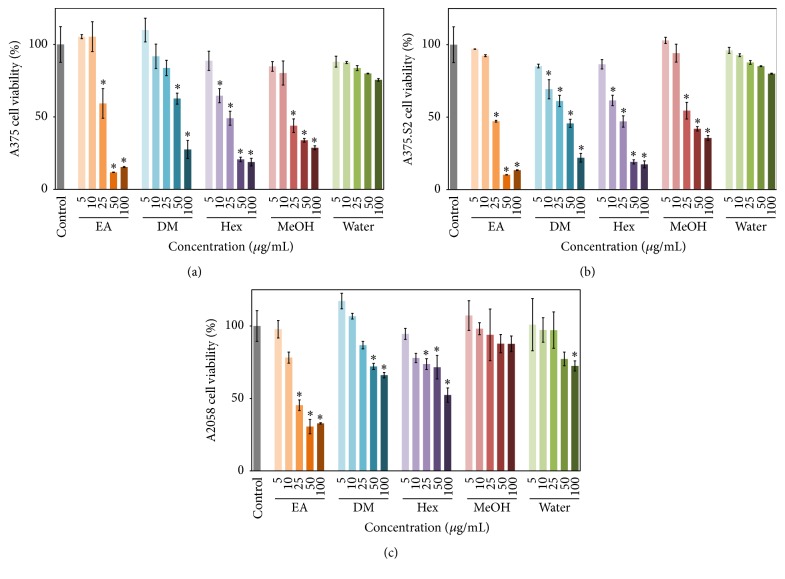
Effects of* E. ramosissimum* extracts on viability of human melanoma cells according to MTT assay. Suppression of cell viability was measured in (a) A375, (b) A375.S2, and (c) A2058 cells cultured with 5, 10, 25, 50, and 100 *μ*g/mL EA, DM, Hex, MeOH, and water. The results for the control group cultured without extract were demonstrated on the left (gray line). All experimental data were presented as average values ± SD; *n* = 3; ^*∗*^ *p* < 0.05.

After establishing the fact that the* E. ramosissimum* extracts did not cause major injuries to normal cells, the melanoma platforms were used to investigate their antiproliferative effects. Cell line A375 was from a malignant melanoma of a 54-year-old female; and cell line A375.S2 comprised cells which were differentiated from A375 cells; and cell line A2058 comprised highly invasive melanoma cells [21]. In Figure 2, we demonstrated the cytotoxicities of the three cell lines, notably, and high doses (50 and 100 *μ*g/mL) of the EA, DM, and Hex extracts from* E. ramosissimum* had much larger influences on A375 and A375.S2 cells compared to the A2058 cells. For example, after 24 h treatment with 50 and 100 *μ*g/mL EA extract, the viabilities of the A375 and A375.S2 cells decreased to 30% whereas the viability of the A2058 cells only decreased to 50%. MeOH extract had no effect on the A2058 cells, and the water extract had no apparent cytotoxic effect on the three melanoma cell types. According to our statistical data, the* E. ramosissimum *extracts have little harmful effects on normal skin cells, and these extracts, particularly EA, DM, and Hex extracts, actually inhibit melanoma cellular proliferation.

### 3.3. Effects of EA and DM Extracts on Caspase Proteins in Melanoma Cells

Caspase is a family of cysteine-aspartic proteases that mediates type I programmed cell death (apoptosis), and more than 10 family members have been identified so far [22]. The activation of caspase-associated proteins is essential for apoptosis induced by various apoptotic stimuli. These changes include blebbing, cell shrinkage, nuclear fragmentation, chromatin condensation, and chromosomal DNA fragmentation; however, the failure of cancer cell apoptosis is a major contributor to tumor development and autoimmune disease [23].

**Figure 3:**
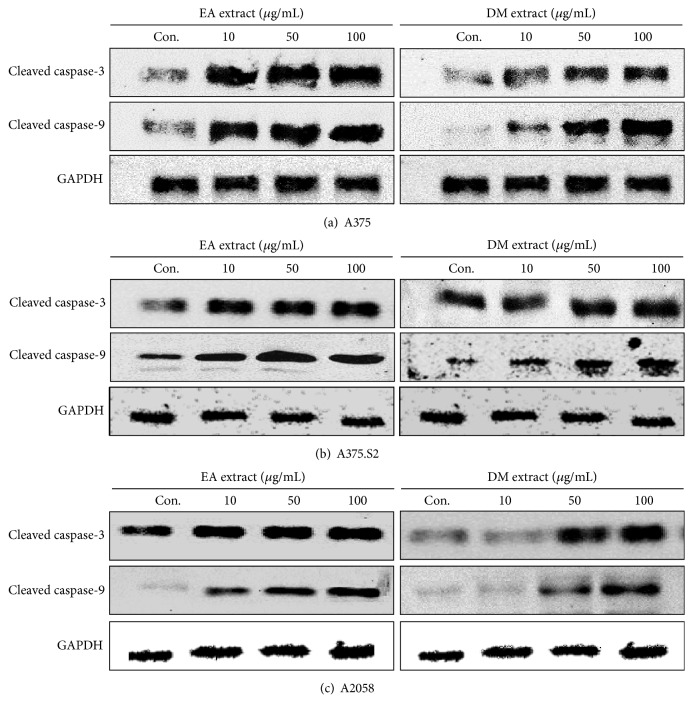
Expressions of caspase proteins in (a) A375, (b) A375.S2, and (c) A2058 cells after treatment with 10, 50, and 100 *μ*g/mL EA and DM extracts for 24 h. Band darkness indicated relative protein expressions in comparison with GAPDH.

Caspase-9 initiates an apoptotic cascade by cleaving and activates caspase-3 [24]. However, the maturation of caspase-9 requires autocatalytic cleavage by apoptosomes released by damaged mitochondria [25]. The cleavage of caspase-3 activates caspase-6 and caspase-7; the protein itself is processed and activated by caspase-8, caspase-9, and caspase-10. The activation of caspase-3 induces cellular apoptosis and proteolysis in specific substrates [26].  Figure 3 showed how caspase affected the apoptotic process induced by EA and DM extracts of* E. ramosissimum* in the three human melanoma cells. Remarkable alterations caused by molecular proteins associated with apoptosis included increased proteolysis induced by caspase-3 and caspase-9. A low concentration (10 *μ*g/mL) of EA extract induced stimulated enzymes in A375, A375.S2, and A2058 cells. Although DM extract also triggered caspase-3 and caspase-9, its effect was smaller compared to a similar dose of EA extract. Notably, 50 *μ*g/mL DM extract was needed to trigger caspase-3 and caspase-9 in A2058 cells. The cellular proteins caspase-3 and caspase-9 have important roles in the regulation of nuclear DNA damage caused by apoptosis and in the decomposition of organelles. Our experiments demonstrated that EA induced caspase protein changes, and similar results were also shown from DM extract, but lower.

### 3.4. Mushroom Tyrosinase Inhibition

In mammals, the rate-limiting enzyme tyrosinase is the most important enzyme of pigment biosynthesis reactions. Other enzymes only adjust to differences in synthesis of eumelanin and pheomelanin [10, 27, 28]. To determine whether the extracts inhibited melanin synthesis by suppressing tyrosinase,* in vitro* tyrosinase activity was tested in mushroom type. In Table 2, it was shown that all extracts except for water had dose-dependent (5–100 *μ*g/mL) inhibiting effects on the mushroom tyrosinase system. At a concentration of 100 *μ*g/mL, the repressive effect of EA extract was slightly lower than that of kojic acid. At this concentration, EA had the strongest inhibiting effect (approximately 40%).

### 3.5. Melanin Content of B16-F10 Cells

**Figure 4:**
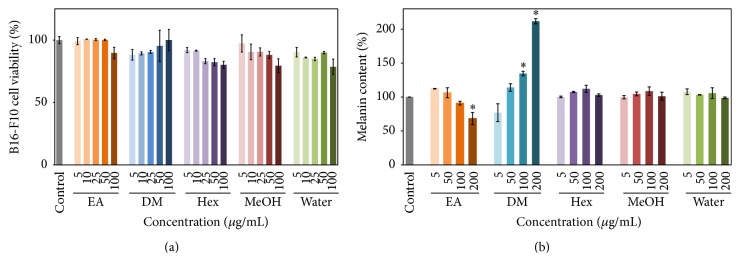
(a) Effects of* E. ramosissimum* extracts on viability of B16-F10 cells according to MTT assay and (b) effects of* E. ramosissimum* extracts on melanin content quantification of all extracts were processed with 5, 50, 100, and 200 *μ*g/mL, respectively. The results for the control group cultured without extracts were shown on the left (gray line). All experimental results were presented as average values ± SD; *n* = 3; ^*∗*^ *p* < 0.05.

Melanin is the source of skin color and can protect the skin from UV radiation damage which induces DNA mutations. Despite its protective functions, superabundance of melanin negatively affects the skin, which then causes social problems [29, 30]. To demonstrate the skin whitening effects of* E. ramosissimum* and its inhibiting effects on melanogenesis, melanin alterations were measured after treatments with* E. ramosissimum *extracts. In Figure 4(a), it was demonstrated that the five fraction extracts did not substantially harm B16 cell viability. In Figure 4(b), we presented the notion that the EA extract had the ability to decrease melanin production by about 32% at 200 *µ*g/mL in a dose-dependent trend from concentrations of 5 to 200 *µ*g/mL. In contrast, 200 *μ*g/mL DM extract substantially augmented melanin production (112%), and the effects of DM extract were dose-dependent. Other extracts, including Hex, MeOH, and water, did not substantially change melanin production, even at the maximum experimental dose of 200 *μ*g/mL. These experimental results suggested that the EA extract had potential applications as a whitening agent in cosmetic products whereas the DM extract could be used as a skin darkening agent.

### 3.6. Expression of Melanogenesis-Related Proteins in B16-F10 Cells

**Figure 5:**
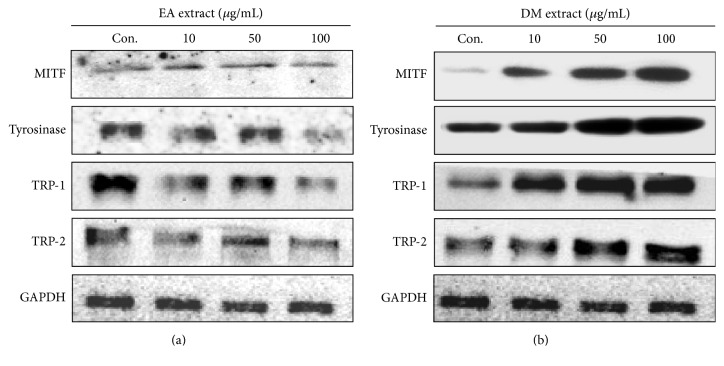
Expressions of tyrosinase, TRP-1, TRP-2, and MITF after treatment with* E. ramosissimum* extracts. B16-F10 cells were treated with EA and DM extracts at concentrations of 10, 50, and 100 *μ*g/mL for 24 h. Protein expressions were shown in comparison with GAPDH.

A well known role of microphthalmia-associated transcription factor (MITF) in melanogenesis is normal synthesis of melanin in melanocytes. Expression of MITF increases melanin production by facilitating biosynthesis of tyrosinase, tyrosinase-related protein 1 (TRP-1), and TRP-2 [31]. Expression of MITF is also significant for stabilizing tyrosinase protein and modulating its catalytic activity [8, 32]. To elucidate the mechanisms of its activity, expressions of melanogenesis-related proteins were analyzed by western blot.  Figure 5 presented the notion that EA extract concentrations ranging from 10 to 100 *μ*g/mL reduced expressions of MITF, tyrosinase, TRP-1, and TRP-2 in a dose-dependent manner. Conversely, DM extract promoted expressions of MITF, tyrosinase, TRP-1, and TRP-2 in B16-F10 cells in a dose-dependent manner. Both of these phenomena were consistent with the data for melanin content shown in Figure 4(b).

## 4. Conclusion

**Figure 6:**
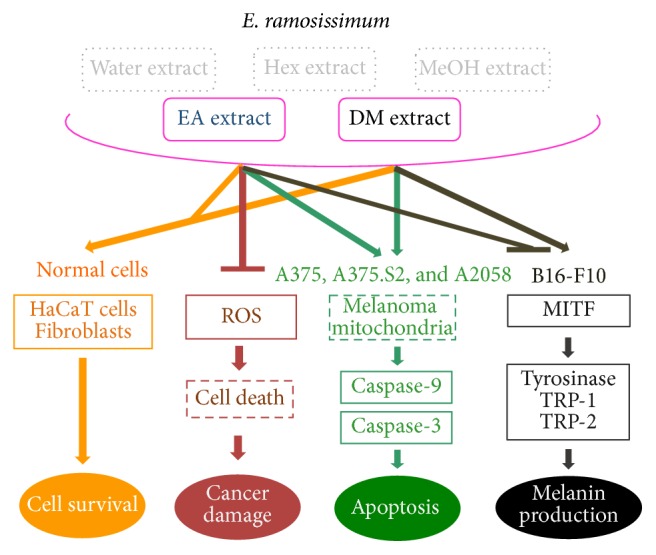
Schematic diagram of biofunctions of* E. ramosissimum* extracts in human skin cells, including normal cell survival, apoptotic pathways of melanoma, and melanogenesis.

In summary, the experiments in this study demonstrated that the most beneficial of the five fraction extracts of* E. ramosissimum *was EA because of its multiple biofunctional properties (Figure 6). The experimental outcomes showed that, by acting as an antioxidant ingredient and electron donor, EA extract discontinued or terminated free radical chain reactions. In human melanoma, EA and DM extracts affected the viabilities of melanoma cells and showed low toxicity in both normal human cells, HaCaT cells and fibroblasts. To understand the mechanisms of cell death, we performed western blot analyses of protein expressions in melanoma cells, which pointed out that both extracts induced caspase-3 and caspase-9, both of which have vital roles in apoptosis. Research evaluations of the potential use of EA extract as a whitening agent illustrated that it inhibited mushroom tyrosinase activity and the synthesis of melanin; in contrast, DM extract increased the quantity of melanin. Western blot analyses showed that EA and DM extracts decreased and increased melanin content, respectively, by regulating MITF, tyrosinase, Trp-1, and Trp-2. Whereas this study established the biological functions of* E. ramosissimum, *our future studies will further investigate the components and mechanisms of these compounds.

## Figures and Tables

**Table 1:** Antioxidant activities of *E. ramosissimum* extracts, including reducing power, DPPH free radical scavenging activity, and ferrous ion chelating power.

Extracts (*μ*g/mL)	Reducing power (OD_700_)			DPPH scavenging capacity (%)			Chelating activity (%)		
	50	100	200	50	100	200	50	100	200
EA	0.60 ± 0.06	0.79 ± 0.06	0.87 ± 0.23	<10.0	15.23 ± 2.76	43.41 ± 7.68	20.47 ± 3.57	34.69 ± 5.03	44.56 ± 1.32
DM	0.59 ± 0.03	0.65 ± 0.07	0.68 ± 0.04	<10.0	<10.0	<10.0	15.57 ± 1.39	17.49 ± 0.13	20.19 ± 1.72
Hex	0.56 ± 0.11	0.65 ± 0.10	0.68 ± 0.03	<10.0	<10.0	<10.0	17.98 ± 4.50	20.71 ± 0.33	22.53 ± 6.07
MeOH	0.58 ± 0.01	0.67 ± 0.13	0.73 ± 0.02	<10.0	10.69 ± 1.66	15.60 ± 1.28	10.63 ± 1.65	18.89 ± 1.19	20.43 ± 0.07
Water	0.62 ± 0.03	0.65 ± 0.10	0.70 ± 0.01	<10.0	<10.0	14.87 ± 0.73	10.77 ± 0.79	13.33 ± 4.55	13.52 ± 0.59
Vitamin C^a^	—	—	—	—	85.55 ± 0.48	—	—	—	—
BHA^b^	—	1.64 ± 0.29	—	—	—	—	—	—	—
EDTA^c^	—	—	—	—	—	—	—	95.49 ± 0.05	—

All statistics are presented as average values ± SD; *n* = 3. ^a^Vitamin C (100 mM) was utilized as a positive control for DPPH assay; ^b^EDTA (100 mM) was used as a positive control for analysis of metal chelating ability; ^c^BHA (100 mM) was applied as a positive control for analysis of reducing power. Assays not performed in this study were indicated by dashes.

**Table 2:** Mushroom tyrosinase inhibition by different concentrations of *E. ramosissimum* extracts.

Concentrations (*μ*g/mL)	Mushroom tyrosinase inhibition (%)		
	5	50	100
EA	22.42 ± 0.25	24.40 ± 4.90	38.93 ± 3.09
DM	20.69 ± 4.53	21.30 ± 1.10	23.79 ± 3.84
Hex	20.54 ± 0.64	22.33 ± 0.69	23.82 ± 4.25
MeOH	18.54 ± 2.70	20.45 ± 0.36	23.27 ± 1.67
Water	17.88 ± 3.46	19.37 ± 0.12	23.82 ± 1.24
Kojic acid^a^	—	—	30.23 ± 5.68

All statistics are presented as average values ± SD; *n* = 3. ^a^Kojic acid (100 *μ*g/mL) was applied as a positive control. Assays not performed in this study were indicated by dashes.
